# Overactive bone morphogenetic protein signaling in heterotopic ossification and Duchenne muscular dystrophy

**DOI:** 10.1007/s00018-012-1054-x

**Published:** 2012-07-04

**Authors:** SongTing Shi, David J. J. de Gorter, Willem M. H. Hoogaars, Peter A. C. ’t Hoen, Peter ten Dijke

**Affiliations:** 1 Department of Molecular Cell Biology and Centre for Biomedical Genetics, Leiden University Medical Centre, Building 2, Room R-02-022, Postzone S-1-P, PO Box 9600, Einthovenweg 20, 2300 RC Leiden, The Netherlands; 2 Institute for Molecular Cell Biology, University of Münster, Schlossplatz 5, 48149 Münster, Germany; 3 The Center for Human and Clinical Genetics, Leiden University Medical Center, Leiden, The Netherlands

**Keywords:** ALK, bone morphogenetic proteins, Heterotopic ossification, Fibrodysplasia ossificans progressiva, Duchenne muscular dystrophy

## Abstract

Bone morphogenetic proteins (BMPs) are important extracellular cytokines that play critical roles in embryogenesis and tissue homeostasis. BMPs signal via transmembrane type I and type II serine/threonine kinase receptors and intracellular Smad effector proteins. BMP signaling is precisely regulated and perturbation of BMP signaling is connected to multiple diseases, including musculoskeletal diseases. In this review, we will summarize the recent progress in elucidation of BMP signal transduction, how overactive BMP signaling is involved in the pathogenesis of heterotopic ossification and Duchenne muscular dystrophy, and discuss possible therapeutic strategies for treatment of these diseases.

## Introduction

Bone morphogenetic proteins (BMPs) were first discovered and described by Marshall Urist as secreted proteins that guide proliferation and differentiation of mesenchymal cells of muscle into bone cells [1, 2]. Nowadays, BMPs are recognized to be multi-functional growth factors that belong to the transforming growth factor β (TGFβ) superfamily, which also includes TGFβs, growth and differentiation factors, activins and Müllerian inhibiting substance. All TGFβ family members are structurally related and are produced by cells as larger precursor proteins that are proteolytically processed into amino-terminal remnants and mature carboxy-terminal parts that bind to cell surface receptors. The mature parts have a characteristic cysteine knot structure. The TGFβ/BMP signaling pathway is essential for orchestration of embryonic development and maintenance of tissue homeostasis in adult animals [3].

More than 20 BMPs have been identified and characterized. Although BMPs were initially identified for their ability to induce bone formation [4], not all of the BMPs appear to be osteo-inductive. On the basis of phylogenetic analysis and sequence similarities, the osteo-inducing BMPs can be divided into three subgroups: the BMP2/4 subgroup, the BMP5/6/7/8 (OP) subgroup and the BMP9/10 subgroup [5]. All of the bone-inducing BMPs can induce mesenchymal stem cells to differentiate into osteoblasts in vitro [5]. However, studies using transgenic and knockout mice or animals with naturally occurring mutations in bone-inducing BMPs suggested that osteo-inductive BMPs are not only necessary for bone and cartilage formation but also play vital roles in heart and neural development (Table 1).

**Table 1:** Osteo-inductive BMPs and its mouse mutant

BMP	Tissue expression	Knockout mice phenotype	Ref.
BMP2	Heart, limb, teeth, muscle	Embryonic lethal, defect in heart development. Conditional knock out in limb showed that BMP2 is dispensable for skeleton formation, but required for bone fracture repair	[151, 183, 184, 185]
BMP4	Teeth, limb, heart, muscle	Embryonic lethal; Little or no mesoderm formation. Conditional knock out of BMP4 showed defects in bone formation	[185]
BMP5	Bone, cartilage	Spontaneous mutation, viable, short ear with skeleton defect, Loss of one pair of ribs	[186]
BMP6	Liver, heart, bone	BMP6 knockout mice are viable, association with type II diabetes and iron overload	[11]
BMP7	Limb, kidney	Die after birth with defects in kidney, eye, and bone	[187]
BMP8	Developing skeleton tissue, male germ cells	BMP8a knockout mice showed defects in maintenance of spermatogenesis, mice deficient in BMP8b are sterile	[9, 188]
BMP9	Liver	N/A	[189]
BMP10	Trabecular myocardium, embryonic and postnatal heart	Embryonic lethal with defects in heart development	[8, 62]

BMPs are morphogens and can induce different cell fates at different concentrations [6, 7]. They are not only required for establishment of dorsal–ventral pattern in embryogenesis but elicit a broad spectrum of biological activities in large variety of tissues, such as repair of bone fracture, maintenance of iron homeostasis, and so on [6–12]. Therefore, BMP signaling needs to be carefully regulated by positive and negative regulatory mechanisms to regulate the intensity and duration of the signaling response in a spatially controlled manner [13]. Perturbations of BMP signaling pathways contribute to progression of a variety of diseases including skeletal diseases, vascular diseases, tissue dystrophy, and cancer [5, 14]. This review will focus on the BMP signaling pathway in general and two different diseases that are linked with ectopic activity of BMPs, i.e., heterotopic ossifications (HO) and the muscle degeneration disease Duchenne muscular dystrophy (DMD).

## BMP receptor signal pathway

### BMP signaling pathway

#### Structure of BMPs and type I and II receptors

BMPs are structurally related cytokines that are found in all multi-cellular organisms. The crystal structure confirmed that the monomers of BMP7 and BMP2 share a common scaffold [15, 16]. Functional studies show that BMPs are highly conserved in evolution; in fact, Decapentaplegic (Dpp) and 60A, the *Drosophila* homologues of BMP2 and BMP7, were shown to induce bone formation in mammals [17] and human BMP4 can rescue dpp null dorsal–ventral patterning in *Drosophila* embryos [18]. Mature BMPs are dimeric proteins that can function either as homodimeric or heterodimeric complexes [19]. Most current knowledge of BMPs are based on studies from homodimeric BMPs, however, both homodimeric BMPs and heterodimeric BMPs are present in vivo, and exert multiple bio-functions [20].

**Fig. 1:**
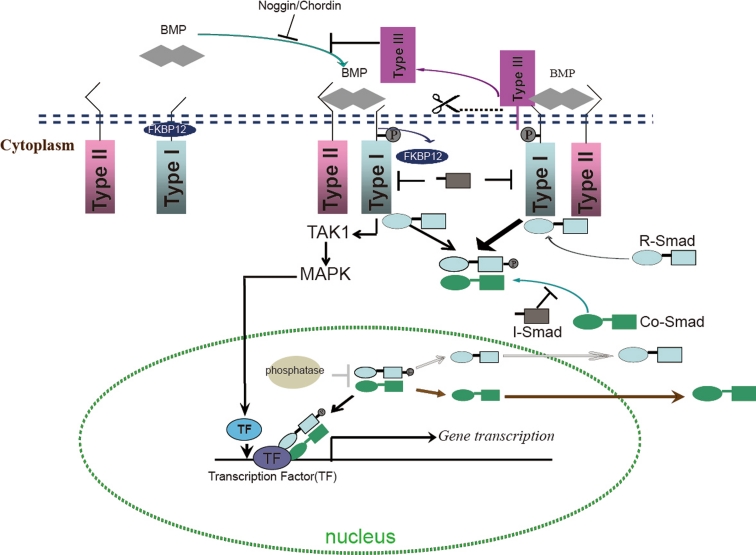
Schematic overview of BMP signaling. Upon formation of heteromeric complex composed of type II and type I receptors and the BMP dimers, FKBP12 is released from the type I receptors and released the phosphorylation site on type I receptor. Next, the type I receptor is phosphorylated by the type II receptor, which propagates the signal into the cells by phosphorylating the C-terminus of R-Smads. The phosphorylated R-Smads form a complex with the Co-Smad and are translocated into nucleus where they in collaboration with other transcription factors to regulate gene expression. The presence of membrane-tethered type III receptors on the membrane can enhance R-Smads phosphorylation. The cells can release the extracellular domain of the type III receptor, which is called the soluble form of type III receptors. The soluble form of type III receptors and other BMP antagonists such as Noggin and Chordin, repress BMP signaling through prohibiting BMP binding to its receptors. I-Smads repress BMP activity either by repressing complex of R-Smads/Co-Smads, or directly inactivate type I receptor activity. In the nucleus, phosphatases represses BMP activity by dephosphorylating R-Smads thereby promoting the exportation of R-Smads. In addition to R-Smads, BMP can also signal via MAPK (non-canonical BMP pathways) through activation of TAK1, which can further activate MAPK. MAPK will be transported into the nucleus, and activate some transcriptional factors, which can further initiate specific gene expression

Like other members in the TGFβ family, BMPs signal across the plasma membrane by interacting and inducing complexes composed of type I and type II receptors that are endowed with intrinsic serine/threonine kinase activity (Fig. 1). In mammals, there are seven type I receptors, the BMPR-I group (ALK3 and ALK6), the ALK-I group (ALK1 and ALK2) and the TβR-I group (ALK4, ALK5 and ALK7) [5]. ALK1, -2, -3, and -6 have been shown to serve as BMP type I receptors. There are four type II receptors in mammals, i.e., BMPR-II, ActR-II and ActR-IIB and MISR-II, of which BMPR-II, ActR-II and ActR-IIB can serve as type II receptor for BMPs that are expressed in multiple tissues [5].

Both type I and type II receptors are required for signal transduction [21]. The type II receptors are constitutively active and are responsible for activating type I receptors. The type I receptor contains a so-called L45 loop that extends from the kinase domain and which is required for interaction and activation of downstream receptor regulated Smads (R-Smads) [5]. The intracellular GS domain (glycine and serine-rich domain) of type I receptors located N-terminal to the serine-threonine kinase domain controls the kinase activity of type I receptors. The phosphorylation of serine and threonine residues in the GS domain by type II receptor activates the kinase activity of the type I receptor and initiates signal transduction mediated by the type I receptor [5]. Under normal circumstances, type I receptors can form oligomeric complexes with type II receptors in the absence of ligands. To prevent type I receptor activation independent of ligand stimulation, the negative regulator FKBP12 binds to the intracellular GS domain of type I receptors thereby preventing it from being phosphorylated in the absence of a ligand [22–24]. Upon ligand stimulation, FKBP12 dissociates from the type I receptors, thereby allowing the phosphorylation by type II receptors on serine and threonine residues in the GS domains. Mutations in the GS domain of type I receptors can lead to constitutive activation of the type I receptors [23, 25]. Notably, in contrast to other type II receptors, the BMPR-II contains a long C-terminal tail following the serine/threonine kinase domain [26]. The C-terminal tail is not involved in BMP-induced Smad signaling, however, in patients suffering from primary pulmonary hypertension (PPH), the C-terminal tail of BMPR-II was found to be truncated, suggesting a unique role for BMPR-II in Smad-independent signaling [27, 28]. Further studies revealed that BMPR-II through its long C-terminal tail mediates BMP-controlled cytoskeletal rearrangements [29, 30].

#### Smad protein-mediated BMP signaling

Upon formation and subsequent activation of a BMP ligand-receptor complex, the activated type I receptors phosphorylate receptor regulated Smad proteins (R-Smads) at their two C-terminal serine residues. ALK1, -2, -3, and -6 mediate the phosphorylation of R-Smad1, -5, and -8. The phosphorylated R-Smads can form complexes with the common mediated Smad (Co-Smad), Smad4, and translocate into the nucleus. In the nucleus, this Smad complex binds the DNA and in collaboration with co-activators and repressors and other transcription factors regulates the expression of specific genes [27].

Recently, the nuclear localized Smads were found to promote also miRNA maturation [31]. TGFβ and BMP stimulation promotes a rapid increase in expression of mature miR-21 through a post-transcriptional step; this process involves R-Smads but is Smad4 independent [31]. The Smad binding sequence on pri-miRNA, which is similar to the Smad binding element (SBE) normally present in the promoter region of TGFβ or BMP responsive genes, is required for R-Smads function in promoting mature miRNA processing [32].

#### Non-Smad BMP signaling

In addition to Smad proteins, BMPs are also able to transduce signals via Smad-independent signaling pathways, for example via ERK, p38, and JNK MAP kinases, small GTPases, and PI3K–Akt/PKPB pathways. BMPs can also activate TGFβ-activated kinase-1 (TAK1), a member of the MAP kinase kinase kinase family [33], which mediates the phosphorylation of p38, JNKs or ERK1/2 in various cell types [34–36]. Interestingly, the ERK1/2 MAPK kinase and TAK1 are important BMP-Smad signaling modulators. It has been demonstrated that both the Smad pathway and the P38/ERK MAPK pathway are required for BMP-induced osteoblast differentiation [37–39]. TAK1 was also shown as positive and negative regulator for Smad signaling. TAK1 was originally discovered as a BMP agonist that synergizes with Smad1/5 to induce ventralization in *Xenopus* embryos [40], however, TAK1 was also shown to interact with R-Smads and to interfere with R-Smads transactivation thereby repressing BMP-induced osteoblast differentiation [41]. Recently, TAK1 was found to promote Smad1/5/8 phosphorylation at C-terminal serine residues in chondrocytes and to be an essential regulator for BMP signaling in chondrogenesis in vitro and in vivo [42–44]. Therefore, BMP-induced TAK1 and its downstream MAP kinases might function as modulators for the canonical BMP-Smad pathway.

#### Modulators of the BMP signaling pathway

Given the important role BMP signaling is playing in a wide variety of biological processes, it has to be tightly regulated. This is achieved by both positive and negative regulation, which occurs at each step of the BMP/Smad signaling pathway. The expression, localization, and activation of BMP ligands, receptors, and Smads are intricately regulated, and this also involves the crosstalk with other signaling pathways [27, 39]. For instance, the Wnt, Notch, and FGF signaling pathways are reported either to be required or to promote BMP-induced osteoblast differentiation [39]. In the following section, we will first discuss the regulation of BMP/Smad signaling at the extracellular level, followed by intracellular BMP receptor/Smad-initiated responses and then the activity of Smads as nuclear effectors.

#### BMP antagonists

Numerous secreted proteins have been identified as BMP antagonists. BMP antagonists can directly bind to BMPs and thereby prohibit BMPs from binding to their receptors. All of these BMP antagonists have a cysteine-knot structure. Based on the size of cysteine-knots, the BMP antagonists can be divided into three subfamilies: the DAN subfamily (eight-membered ring) including USAG-1 and Sclerostin, the twisted gastrulation (Tsg) subfamily (nine-membered ring), and chordin and Noggin (ten-membered ring) [45, 46]. Detailed functional studies demonstrated that BMP antagonists selectively block the activity of specific BMPs. For instance, Noggin inhibits BMP2 and BMP4 but can not block BMP6 and BMP9 activity [47]. Chordin can bind to BMP2, -4, -7, but cannot interact with the other BMP-family proteins [48]. Sclerostin binds BMP6 and BMP7 and inhibits their activity [49].

#### BMP co-receptors

Currently a number of transmembrane and membrane-anchored proteins have been characterized as co-receptors or type III receptors, and function as modulators for TGFβ/BMP signaling. The repulsive guidance molecules (RGM) family, comprised of RGMa (also known as RGM), RGMb (also known as Dragon), RGMc (also denoted as Hfe2 or HJV), and RGMd (currently only found in fish [50]) form the first known BMP selective co-receptor family that can potentiate BMP signaling. RGM proteins are glycosylphosphatidylinositol (GPI)-anchored membrane proteins [50–53]. The mechanisms of how RGM proteins potentiate BMP signaling are still elusive. One possibility is that RGM proteins can interact with type I receptors and alter utilization of BMP type II receptors by BMP ligands [52, 54]. Recently, RGMs were suggested to enable association of neogenin with other BMP receptors (BMPRs) in lipid rafts of chondrocytes. The modes of receptors oligomerization could determine which downstream BMP signaling pathways are activated [55]. Neogenin and RGMc could facilitate the formation of membrane receptor complexes that deliver continuous Smad signaling, and are required for BMP-induced chondrogenesis in vitro and in vivo [56]. Betaglycan and endoglin were initially described as co-receptors for TGFβ, but have also been shown to function as co-receptors for BMP2, -4, -7, and BMP9, respectively [57–59]. Betaglycan can promote the binding of BMP ligands, BMP2, -4, -7, to ALK3 and ALK6 to enhance BMP signaling [57]. Endoglin is highly expressed in endothelial cells, which usually have little or no expression of betaglycan [60, 61]. The presence of endoglin in the endothelial cells may make endothelial cells more responsive to ALK1-mediated BMP9 signaling [59, 62]. Notably, cells can release the extracellular domain of the co-receptors by cleavage at the sites near their transmembrane regions [63–65]. The soluble forms of RGM proteins and endoglin, which only contain the extracellular domain of these receptors, were established as BMP inhibitors in recent studies [11, 66] as they could possibly compete with membrane-receptors for binding to BMP ligands. Moreover, *Xenopus* BAMBI and its mammalian homolog Nma have been identified as pseudo-receptors that contain extracellular domains structurally related to that of type I receptors. They lack the intracellular kinase domain, and as a consequence function as repressors of BMP signaling to prevent the formation of active receptor complexes [67, 68].

#### Intracellular regulation of BMP signaling

The inhibitory Smads (I-Smad) comprise Smad6 and Smad7, which serve as inhibitors for the Smad signaling pathways. Smad6 mainly targets BMP signaling while Smad7 represses both TGFβ and BMP signaling [69–71]. Smad6 has been shown to compete with R-Smads for interaction with Smad4, and can recruit the transcriptional corepressor CtBP to repress BMP-induced transcription [69, 72]. Smad7 represses R-Smad phosphorylation. In addition, it can recruit phosphatases that can mediate type I receptor dephosphorylation and inactivation [70, 73].

The ubiquitin system also actively participates in the regulation of BMP signaling. Smad7 can recruit Smurf E3 ubiquitin ligases and mediate the turnover of activated type I receptors [74, 75]. In addition, Smurfs can directly interact with R-Smads and promote their degradation [76, 77]. The deubiquitinating enzyme (DUB) UCH37 can bind to Smad7. Then it can deubiquitinate and stabilize type I receptors and hence function as agonist for TGFβ signaling [78].

Smad anchor for receptor activation (SARA) protein can enhance TGFβ signaling by recruiting and presenting non-phosphorylated R-Smads to active membrane type I receptors [79]. Recently, endofin was characterized as a protein acting similarly to SARA in BMP signaling, which can recruit nonphosphorylated Smad1, enhance Smad1 phosphorylation, and the subsequent nuclear translocation of Smad1 [80].

Since the BMP signaling is transduced by phosphorylated C-terminal R-Smads, phosphatases for R-Smads could function as repressor for BMP signaling. The phosphatase proteins, small C-terminal domain phosphatase (SCP1/2) and protein phosphatase magnesium-dependent 1A (PPM1A), can efficiently dephosphorylate the C-terminal domain of Smad1 in the nucleus and attenuate BMP signaling [81, 82]. Recently, SCP1 was established to repress BMP-induced osteoblast differentiation [83]. In contrast, PP2A, which can dephosphorylate R-Smads at their linker region, was shown to enhance canonical Smad signaling [84].

In the nucleus, the activated Smad complexes interact with other transcription factors to control gene expression. Histone deacetylases (HDACs), chromatin modulators, function as negative regulators for BMP signaling. c-Ski and Twist-1 are well-studied negative regulators for BMP signaling, which interact with Smad4 and recruit HDACs to the Smad complex thereby repressing its transcriptional activity and antagonize BMP signaling. Both c-Ski and Twist-1 can inhibit BMP-induced osteoblast differentiation [85–88].

#### Endocytosis and BMP signaling

Formation of the ligand-receptor complex can initiate endocytosis of active ligands and receptors. Endocytosis-mediated internalization of receptors cannot only control receptor density, thus modulating signaling activity, but is also required for signal transduction in some situations [89, 90].

It was proposed that BMP type I receptors internalization is mediated by clathrin-mediated endocytosis, which is required for continuation of Smad signaling [91]. Endofin, the SARA-like protein in BMP signaling located in the endosome derived from clathrin-coated pits, promotes the BMP-Smad signaling [80]. However, the interaction of Smad7–Smurf2 complexes that are present in lipid raft caveolae to the type I receptors can promote their rapid turnover and repress signaling [89].

#### Negative feedback loops for BMP signaling

In the previous section, we have discussed various negative regulatory mechanisms for BMP signaling. Multiple negative regulators have been demonstrated to be direct target genes of BMP signaling. For instance, Noggin/Chordin and I-Smads are all well established as direct BMP target genes [92–96]. Endofin contains a protein-phosphatase-binding motif. Depending on the amount of nonphosphorylated Smad1 in the cells, endofin can also function as a BMP inhibitor by recruiting phosphatases to inactivate type I receptors [80]. The activation of BMP signaling can also result in degradation of type I receptors via endocytosis [89]. These mechanisms establish auto-regulatory negative feedback loops for BMP signaling to exert spatial–temporal control over its multiple activities.

In the above sections, we have summarized current research results on BMP signaling. BMPs were originally discovered as bone inducers and repressors of myogenesis [1, 4, 97]. In the following sections, we will discuss the roles of BMP signaling in the progression of two representative bone and skeletal muscle diseases: heterotopic ossification (HO) and Duchenne muscular dystrophy (DMD).

## BMP signaling in heterotopic ossification

Heterotopic ossification (HO) is defined as bone formation at aberrant locations outside the skeleton; mature bone tissue can be found in the soft tissue where bone normally does not exist. The presence of HO might cause joint stiffness, limited range of motion, swelling and pain, and can even result in severe functional limitations [98]. HO was first clearly described in 1883, and then in 1918, Déjerine and Ceillier found that soldiers in World War I with spinal cord trauma frequently acquired HO. Nowadays, it is well described in multiple clinical reports, for example, patients who have total hip arthroplasty or injury at spinal cord are at risk of developing HO [99]. A few years ago, Charmers and colleagues proposed that osteogenic precursor cells, inducing agents and permissive microenvironments are essential conditions for ectopic bone formation [100]. Among all the discovered osteo-inductive growth factors, BMPs are considered important growth factors involved in bone formation; the ability to induce bone when implanted at ectopic sites in rats led to their discovery [1, 4, 101–103]. Besides the trauma-induced HO, there is also a hereditary form of HO called fibrodysplasia ossificans progressiva (FOP).

### Fibrodysplasia ossificans progressiva

Fibrodysplasia ossificans progressiva (FOP) has an incidence of 1 in 2 million. Patients develop progressive heterotopic ossification (HO) in the soft tissues either as a result of trauma or spontaneously. Children born with FOP appear normal at birth apart from deformed great toes [25]. Before the age of ten, FOP patients develop painful and highly inflammatory soft tissue swellings, which can transform into bone [104]. The occurrence of ectopic bone usually follows a fixed pattern: starting from the neck, then in the shoulders, arms, chest areas, and finally in the feet. The development of ectopic bone formation in FOP patients occurs through an endochondral ossification pathway. A histological examination identified several stages of the FOP lesion formation: lymphocyte infiltration, degradation of muscle tissue, fibroproliferative and highly angiogenic stages, cartilage and finally formation of bone [105]. Minor trauma to soft tissue can initiate painful ectopic bone formation in FOP patients, but sometimes bone formation seems to occur spontaneously without detectable trauma [106, 107]. Surgical resection to remove the ectopic bone tissue is not an option for treatment of the FOP patients as the surgical trauma induces the formation of new heterotopic bone [107].

In 1997, Shafritz reported that BMP4 is overexpressed in lymphoblastoid cells and lesional cells of FOP patients [108]. The BMP4 antagonist Noggin is a direct target gene for BMP signaling. However, BMP4-induced Noggin expression in lymphocytes of FOP patients was found to be attenuated compared to the control lymphocytes [108, 109], implying the dysregulation of BMP4–Noggin negative feedback loop in FOP patients. However, until now, only transgenic mice expressing BMP4 under the control of the neuron-specific enolase (NSE) promoter developed a FOP-like phenotype [102]. Others reported that BMP4 transgenic mice either died at birth or failed to develop a FOP-like disorder [110–113]. Moreover, the FOP lymphocytes displayed higher expression of ALK3 and a defect in endocytosis-dependent degradation of BMP type I receptors, which could result in constitutively high expression of ALK3 on the membrane [114]. In 2006, the gene responsible for the FOP disease was identified as the ALK2 gene encoding a BMP type I receptor. The classic FOP-associated ALK2 mutation is R206H; this residue is located in the GS domain and interferes with the binding of the negative regulator FKBP12, which results in ALK2 activation in the absence of BMP ligands [25, 115–117]. Recently, an ALK2 R206H knock in mice was reported to have FOP symptoms, including malformed first digits in the hind limbs and postnatal extraskeletal bone formation [118]. These results further supported that mutant ALK2, which can sensitize mesenchymal cells to undergo BMP-induced osteoblast differentiation and bone formation in vitro, caused FOP [118].

The mutated ALK2 in FOP patients that leads to elevated BMP signaling plays a pivotal role in ectopic bone formation in the FOP patients. However, transgenic mice with global postnatal expression of constitutively activated (CA)-ALK2 (induced without inflammation) do not develop ectopic bone. CA-ALK2 in combination with local inflammation mediated by adenoviral infection induced bone formation in skeletal muscle, joint fusion, and functional impairment [119]. Moreover, mice treated with the anti-inflammatory drug dexamethasone showed significantly reduced ectopic bone formation induced by adenoviral infection in the skeletal muscle of CA-ALK2 transgenic mice [119]. Many studies on FOP patients support the important role of inflammation in disease progression [120, 121]. A clinical study found that bone-marrow transplantation from a normal donor in a FOP patient, which received immunosuppression, ameliorated the activation of ectopic bone formation [122]. Thus, hematopoietic cells may contribute to ectopic bone formation.

**Fig. 2:**
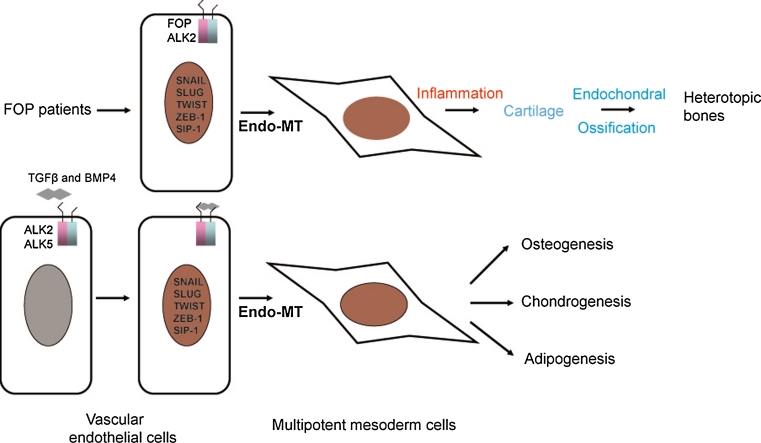
Diagram illustrating endothelial-to-mesenchymal transition (Endo-MT) and its role in the heterotopic bone formation in FOP patients. In the endothelial cells, TGFβ or BMP4 induces expression of transcription factors for mesoderm induction including Snail/Slug/Twist/ZEB-1/Sip-1, and reprogram endothelial cells into multipotent mesoderm cells through Endo-MT transition. The multipotent mesoderm cells can be further differentiated into osteoblasts, chondrocytes, and adipocytes under proper differentiation conditions. The type I receptors ALK2 and ALK5 participate in the process. In FOP patients, the mutant ALK2 can directly induce Endo-MT and convert endothelial cells into multipotent mesoderm cells. Then, under pathogenic inflammatory conditions in FOP patients, the mesoderm cells differentiate into cartilage, which can be further developed into heterotopic bone through endochondral ossification

For a long time, osteoprogenitor cells for ectopic bone were considered to be the mesenchymal stem cells residing in the skeletal muscles which have the potential to differentiate into multiple mesenchymal lineages [101, 123–126]. In 2010, Medici and colleagues [127] showed an endothelial origin of up to 50 % of the heterotopic cartilage and bone in both FOP patients and the CA-ALK2 transgenic FOP mouse model. In vitro, CA-ALK2 or TGFβ and BMP4 stimulation is able to induce endothelial-to-mesenchymal transition (Endo-MT). CA-ALK2 or TGFβ and BMP4 stimulation induce the expression of transcription factors Snail/Slug/Twist/ZEB-1/Sip-1, all of which are important for epithelial-to-mesenchymal (EMT) transition, and convert mature endothelial cells into mesenchymal stem cell-like cells, which subsequently differentiate into chondrocytes, osteoblasts, or adipocytes under the appropriate differentiation conditions [127] (Fig. 2). In addition, the increased number of circulating osteoprogenitor cells of hematopoietic origin, were reported to associate with active HO formation in patients with FOP, and to be present in the pre-osseous fibroproliferative lesions. Therefore, circulating osteoprogenitor cells are another group of osteoprogenitor cells that can contribute to HO in susceptible host tissue [128].

Elevated BMP signaling due to a defect in the auto-regulatory feedback loop was already confirmed in the lymphocytes from FOP patients [129]. Lymphocytes are important responsive cells in inflammation and therefore it could also be interesting to determine the role of lymphocytes with dysregulated BMP activity in inflammation and whether they can promote bone formation. Moreover, the defective negative regulatory loop of BMP signaling is mainly observed in lymphocytes from FOP patients. It is not clear whether the negative regulation mechanism is also defective in osteoprogenitor cells in FOP patients. It could be interesting to investigate negative regulatory mechanisms in the osteoprogenitor cells from the FOP patients, and whether mutated ALK2 could lead to defective negative regulatory mechanisms in lymphocytes and other cell types.

Mutated ALK2 from FOP patients can directly convert mature endothelial cells into multi-potent mesenchymal cells, and sensitize mesenchymal cells to BMP-induced osteoblast differentiation [117, 127], thus making ALK2 a putative therapeutic target to prevent HO in FOP patients. LDN-193189, a specific BMP type I receptor kinase inhibitor, with the highest potency towards ALK2 kinase activity, was reported to decrease HO in CA-ALK2 transgenic mice [119]. Although in CA-ALK2 transgenic mice, LDN-193189 treatment showed no toxicity on mice growth, behavior, or bone density [119], further investigation is required before LDN-193189 can be applied to FOP patients. LDN-193189 is a potent BMP receptor inhibitor that significantly reduces ALK2 activity, but also other kinase activities at high dose [130, 131]. Recently, BMP signaling was found to be required for muscle regeneration, as discussed in more detail below, and therefore complete inhibition of ALK2-dependent BMP signaling should be avoided. The ideal kinase inhibitor for treatment of FOP without possible on-target side-effects in other tissues is one that specifically targets mutated ALK2, inhibiting the extra activity of ALK2 derived from the FOP allele, while also not affecting ALK1, ALK3, ALK6, and wild-type ALK2 kinase activity. Nowadays, genetic tools, including antisense therapy and RNA interference, have already been developed to modify specific gene or protein expression [132, 133]. Kaplan and colleagues have successfully employed allele-specific RNAi (ASP-RNAi) technique to reduce disease-causing ALK2 activity without inhibition of the normal ALK2 allele in FOP cells [134]. In the future, other genetic tools to specifically modulate mutant ALK2 expression in FOP patients might be good alternatives for treatment of disease.

#### Acquired form of heterotopic ossification

As mentioned before, FOP is a very rare genetic disease characterized by progressive heterotopic ossification (HO) induced by an activated ALK2 mutant. The most common form of HO is the acquired form, which is either induced by trauma or linked to damage in the nervous system (spinal cord or brain). Post-traumatic HO is caused by injuries at soft tissue at any site. The most common post-traumatic HO is observed after severe long bone fracture or in the hip after total hip arthroplasty [135]. More than 50 % of patients develop HO after total hip arthroplasty [136, 137]. The damaged muscle is another area at high risk of developing HO [101, 102, 138]. Recently, HO was unexpectedly discovered in end-stage valvular heart disease [139–142]. In spinal cord injured patients, the incidence of HO is between 20 and 25 %, while in closed brain injury HO occurs in 10–20 % of patients [99]. Patients with brain injury could develop peri-articular HO especially in the hip and elbow joint [143]. For patients with spinal cord injury, the HO is mostly observed in the hip region [144]. Until now, it is not well understood how the injuries in the nervous system lead to HO in the hip or elbow joint. In the following section, we will mainly discuss post-traumatic HO.

As mentioned, inflammatory conditions have been reported to be important for the progression of the disease in FOP [120, 145]. Like in FOP, inflammation is involved in the formation of ectopic bone in acquired HO. The pro-inflammatory cytokine, TNFα can stimulate the expression of BMP2, an important bone inducer in endothelial cells [146]. In addition, TNFα can augment the recruitment and differentiation of muscle-residing stroma cells (mrSCs) to enhance bone formation [125]. So the nonsteroidal anti-inflammatory drugs (NSAIDS) are important therapies for reducing the risk of HO. The anti-inflammatory agent Indomethacin is already commonly used as treatment of patients after acetabular fracture to prevent the possible occurrence of HO [147].

BMPs may mediate the induction of acquired HO. In the valve where HO was identified, BMP2 and BMP4 were found to be expressed by myofibroblasts and preosteoblasts in areas adjacent to B- and T-lymphocyte infiltrations [139]. Moreover, multiple studies showed that BMP2, BMP4, and BMP9-induced HO in skeletal muscle by intramuscular injections [101, 103, 138].

Tie2^+^ progenitor cells are discovered as major osteoprogenitor cells that respond to an inflammatory stimulation and further differentiate into heterotopic bones in BMP2 and BMP4-induced HO in the skeletal muscle [138]. Tie2 is a hallmark for endothelium cells, suggesting an important role of endothelial cells in contributing to HO [127, 138]. Interestingly, another report identified a group of non-endothelium Tie2^+^ cells residing in the interstitium of skeletal muscle and other tissues, displaying multi-potent ability to differentiate into mesoderm linage cells including osteoblasts and adipocytes [148]. Instead of other Tie2^+^ cells from the endothelium, it is this Tie2^+^ cell population of non-endothelium origin that are responsive to BMP2-induced HO in the skeletal muscle [148]. Medici et al. [127] discovered that multipotent cells derived from endothelial cells still expressed Tie2. Therefore, it would be interesting to investigate whether these Tie2^+^ multipotent mesenchymal cells might be converted from endothelial cells, or whether the niche holding these cells could facilitate transition of endothelial cells into multipotent mesenchymal cells in vivo. In addition to Tie2^+^ cells, mrSCs also contributed to the HO induced by BMP2 and BMP9 in the damaged muscle [101]. In addition, circulating osteogenic precursor cells are considered as a group of possible osteoprogenitor cells for HO. Circulating osteogenic cells were discovered to home to sites of vascular injury and were associated with HO formation in the heart valve [149].

Interestingly, in an in vitro study, BMP9 appeared to be more potent for inducing differentiation of mrSCs into osteoblasts than BMP2 [101]. In vivo, BMP2 can induce ectopic bone formation in the skeletal muscle with or without cardiotoxin (CTX)-induced muscle damage, whereas BMP9 only induced ectopic bone formation in CTX-induced damaged muscle [101]. Thus, it seems that BMP2 and BMP9 are not functionally equivalent to induce bone formation in the skeletal muscle [101, 150]. In vivo, BMP2 is secreted at the bone fracture area and is required for the initiation of fracture healing [12, 151]. BMP9 appears to function mainly in angiogenesis [152]. Therefore it would be interesting to investigate whether BMP2 and BMP9 play different roles in the initiation of bone formation in the muscle, or have different roles in the inflammatory reaction. A recent study suggested a group of non-endothelial Tie2^+^ cells as osteoprogenitor cells responsive to BMP2 to induce bone formation in skeletal muscle [148]. Further research might focus on the responsiveness of these cells to BMP2 and BMP9 stimulation in normal muscle or CTX injured muscle.

Different from FOP patients, in whom the ectopic bone is impossible to be removed by surgical operation, the only effective treatment of symptomatic established HO is surgical resection of ectopic bone tissue. To prevent the possible relapse of HO, it is prudent to avoid soft-tissue trauma in the operation room. Gentle handling of tissues includes complete wound lavage and removal of all bone debris and reaming was suggested to decrease the risk of HO after surgery [153, 154]. Radiation and usage of NSAIDS are used to further decrease the chance of getting HO [154]. Radiating pluripotential mesenchymal cells, the possible osteoprogenitor cells for HO, may effectively prevent the formation of HO [155]. Unfortunately, the NSAID therapy, while reasonably effective, has side-effects, most notably gastrointestinal ulceration, decreased platelet aggregation and renal toxicity [154, 156]. Since BMPs are well-established inducers of the HO, BMP inhibitors might turn out to be useful in the prevention of HO in the future.

## BMP signaling in muscle regeneration and DMD disease

### BMP signaling in muscle regeneration

In damaged regenerating muscle, BMPs, such as BMP2, BMP4, and BMP9, can potently induce bone formation [138]. Therefore one may think of the use of BMP inhibitors to repress HO in the skeletal muscle. However, recently studies [157, 158] on muscle regeneration after muscle damage suggested an essential role of BMP signaling in muscle regeneration [159].

Muscle regeneration is comprised of three steps: upon muscle damage, quiescent muscle stem cells or satellite cells (characterized by Pax7^+^, MyoD^−^), which reside between the basement membrane and sarcolemma of individual muscle fibers, are activated; activated satellite cells (Pax7^+^, MyoD^+^) proliferate and subsequently either differentiate into myoblasts (Pax7^−^, MyoD^+^, Myog^−^), which initiate myogenic differentiation (Pax7^−^, MyoD^+^, Myog^+^) and fuse to repair damaged fibers or form nascent muscle fibers. Part of the activated satellite cells convert back to quiescent satellite cells (Pax7^+^, MyoD^−^) thereby self-renewing the satellite cell pool [160]. Together with satellite cells, mrSC, fibroblasts, and immune cells also participate in the regeneration process. Following acute tissue injury, inflammatory cells, fibroblasts, and mrSC migrate to the injured areas to restore tissue homeostasis. The inflammatory cells remove the damaged or dead fibers, which are then replaced by the activated satellite cells and the mrSCs [161]. In addition, fibroblasts and inflammatory cells stimulate satellite cell activation by secreting stimulatory growth factors/cytokines, such as HGF, FGF, and IGF [162]. The relevance of these different cell populations in muscle regeneration has recently been shown by several studies. Ablation of satellite cells, muscle fibroblasts, or macrophages all resulted in impaired muscle regeneration in mice [163–166].

Upon muscle damage, ALK3 expression is elevated in the activated satellite cells, and Smad1/5/8 phosphorylation is detected in the nucleus of the activated satellite cells, implying the activation of the BMP pathway [157]. In contrast, BMP antagonist Noggin is expressed in satellite cells committed to myogenic differentiation. The use of BMP inhibitors to repress BMP signaling in the regenerating muscle by means of dorsomorphin or a soluble ALK3 extracellular domain ligand trap, resulted in smaller regenerated myofibers and fibrosis; in vitro either inhibition of Noggin or exogenous BMP4 stimulated satellite cells division and repressed satellite cell differentiation, whereas addition of Noggin or soluble ALK3 had the opposite effect. Therefore, it was hypothesized that BMP signaling is required for the maintenance of the pool of activated satellite cells [157]. Clever’s study on Id1+/−; Id3−/− mutant mice suggested that Id1/3, which are direct target genes of the BMP-induced Smad pathway, mediate BMPs inhibitory effect on muscle differentiation. The Id1+/−; Id3−/− mutant mice displayed delayed and reduced skeletal muscle regeneration, characterized by a decreased number of activated satellite cells after injury [167]. BMP signaling repressed differentiation of satellite cells into myotubes. Therefore, the activity of BMP signaling should be switched off when enough satellite cells have been generated. The mechanisms controlling the timing that induce the satellite cells to switch from proliferation to differentiation are not well deciphered. But BMP antagonist Noggin and Chordin are elevated upon differentiation, which could repress the endogenous BMP signaling in activated satellite cells, and initiate the differentiation program [130, 157, 158].

Activation of BMP signaling is necessary for maintenance of activated satellite cells in the damaged muscle. However, BMP2, BMP4, and BMP9 have been demonstrated to induce ectopic bone formation in the damaged muscle. Therefore one might be prudent when using BMP inhibitors to repress HO occurrence in skeletal muscle as they may disturb the muscle regeneration. One option to overcome this complication could be to use the inhibitors that could specifically target the osteoprogenitor cells for ectopic bone cells. Another possibility is to inhibit the activity of receptors that are involved in the HO process, but not in the muscle regeneration process. Up to now, ALK3 is the only BMP type I receptor discovered to be involved in the muscle regeneration process [157] and it is not known if the other BMP receptors are involved in this process. It is not known whether ALK3 is also actively involved in HO occurrence in the skeletal muscle, like ALK2. Further research should therefore be focused on the specific and/or overlapping functions of different BMP type I receptors in muscle regeneration and HO.

#### BMP signaling in DMD disease

BMP signaling is important in balancing the satellite cell proliferation and differentiation program. Dysregulated BMP signaling might be linked with progression of muscle diseases. DMD disease is one of the diseases in which elevated BMP signaling in the satellite cells might exacerbate the disease [168].

DMD disease is a recessive X-linked form of muscular dystrophy that results in muscle degeneration. The disease is caused by mutations in the *DMD* gene [169], encoding the dystrophin protein that connects the cytoskeleton of muscle fibers to the underlying basal lamina. The absence of functional dystrophin in the myofiber leads to membrane damage, which results in increased calcium-influx and subsequent muscle fiber breakdown in DMD patients [170, 171]. Due to the constitutive muscle fiber damage, DMD patients suffer from chronic inflammation, in which infiltrated inflammatory cells and persistently activated fibroblasts stimulate fibrosis [161]. In addition to fibroblasts, muscle fibers in DMD patients are replaced by adipose tissue, although the underlying molecular mechanism is unknown [172]. Furthermore, the muscle’s regenerative capacity may be exhausted under chronic inflammatory conditions. Although the mechanism is not known, the continuous activation of satellite cells may lead to depletion of the satellite cell population. In addition, myoblasts isolated from DMD patients show proliferation and/or differentiation defects, which may further contribute to the decline in muscle regeneration [173, 174]. Moreover, myoblast to myofibroblast transdifferentiation has been reported to be partially causal for muscle fibrosis and may also further contribute to impaired muscle repair in DMD muscle [175].

To find the underlying molecular mechanism for the inefficient differentiation of DMD myoblasts, Sterrenburg and colleagues performed a microarray assay to compare expression profiles in DMD myoblasts and healthy myoblasts [168]. BMP4 expression was found to be significantly higher expressed in DMD cultures compared to myoblasts of healthy individuals [168]. BMP4 can maintain satellite cells in a proliferative state and inhibit myogenic differentiation [157]. In vitro, BMP4 was shown to inhibit both MyoD and myogenin, muscle-specific transcription factors that regulate differentiation of satellite cells into skeletal muscles. The elevated level of BMP4 in DMD myoblasts could partially explain the inefficiency of satellite cells to form new muscle fibers in DMD patients [176].

Muscle fibrosis is a prominent pathological symptom in DMD patients. The TGFβ signaling pathway has already been established as a key factor involved in fibrosis in DMD patients [177]. Multiple studies have demonstrated that BMP7 can reduce TGFβ-induced renal fibrosis and cardiac fibrosis [178–180]. Recently, BMP6 was discovered to attenuate TGFβ signaling in Dupuytren’s fibroblasts, and inhibit the fibrotic response [181]. However, there are no reports that BMP signaling affects TGFβ-induced muscle fibrosis. It is not known whether elevated BMP4 signaling contributes to the chronic inflammatory reaction in damaged skeletal muscle and subsequent fibrosis in DMD patients. A recent study suggested that BMP4 can induce EndoMT [127]. EndoMT transition has been demonstrated to contribute to cardiac fibrosis [180]. Therefore BMP4 might possibly play a role in muscle fibrosis through induction of EndoMT. It would be interesting to investigate the exact role for BMP signaling in muscle fibrosis.

As mentioned above, adipose tissue also replaces muscle fibers in dystrophic muscle of DMD patients [172]. A group of Tie2^+^ cells residing between skeletal muscle and endothelium was established as multipotent cells, and could differentiate into adipocytes in vitro [148]. BMP4 is able to induce mesenchymal stem cells into adipocyte-lineage cells in vitro [182]. Therefore BMP4 might also contribute to the accumulation of adipocytes in DMD patients.

To validate whether BMP signaling indeed contributes to the progression of DMD disease, we administrated dystrophin-deficient *mdx* mice with the BMP antagonist Noggin [130]. We observed enhanced MyoD and myogenin expression in the mice treated with Noggin, suggesting improved muscle regeneration, characterized by improved muscle histology [130], but could not detect a decrease in the inflammatory response. These results suggested that inhibition of BMP signaling might be beneficial for improving muscle regeneration in DMD patients. However, considering the importance of BMP signaling during muscle regeneration in healthy muscle, a potential beneficial effect of BMP antagonists in dystrophic muscle is likely to be dose-dependent, and complete repression of BMP signaling may even be detrimental. Therefore, the dose-dependent effects of such approaches should be assessed in more detail in animal models of DMD. In addition, one has to keep in mind that improving muscle regeneration will not result in improved muscle function in DMD muscle, since the primary genetic defect remains. Therefore, future DMD therapies should aim at both restoring dystrophin function and improve the muscle condition by counteracting fibrosis and improving muscle regeneration.

## Conclusions

In this review, we have summarized the current progress of research on HO and DMD diseases that are related to elevated BMP signaling. The critical involvement of overactive BMP signaling in ectopic bone formation in HO patients is well established. Whether deregulated BMP signaling also contributes to DMD pathology by repressing muscle regeneration needs more investigation. Inhibition of excessive BMP signaling might be a promising therapeutic approach for treatment of these diseases, especially in FOP patient and DMD patients, in which the surgical treatment is impossible.

BMPs have been well established as crucial cytokines that control multiple biological phenomena, either during embryonic development or to control postnatal tissue homeostasis. Therefore anti-BMP treatment in the above-mentioned diseases should be considered with care to prevent the possible on-target side-effects and dose-dependent effects of such treatments should be determined in animal models. The BMP type I receptor kinase inhibitors dorsomorphin and LDN-193189 have shown to inhibit at high dose both BMP and TGFβ activity. The BMP antagonist Noggin is not so stable in vivo and was shown not to be able to inhibit BMP6 and BMP9 [47]. The soluble receptors and the neutralizing antibodies can only target the extracellular BMPs, therefore they would not be so beneficial for treatment of FOP patients who have mutated ALK2. Currently, ASP-RNAi technique has been successfully applied to specifically decrease mutant ALK2 allele activity and restore normal BMP activity in FOP cells [134]. In the future, other genetic tools, including antisense therapy and mi-RNA can be employed to decrease BMP activity.

## Abbreviations

HO: Heterotopic ossification
DMD: Duchenne muscular dystrophy
BMP: Bone morphogenetic protein
TGFβ: Transforming growth factor-β
Dpp: Decapentaplegic
R-Smad: Receptor regulated Smad
SBE: Smad binding element
TAK1: TGFβ-activated kinase-1
RGM: Repulsive guidance molecule
GPI: Glycosylphosphatidylinositol
I-Smad: Inhibitory Smad
DUB: Deubiquitinating enzyme
SARA: Smad anchor for receptor activation
SCP: Small C-terminal domain phosphatase
PPM1A: Protein phosphatase magnesium-dependent 1A
HDAC: Histone deacetylase
NSE: Neuron-specific enolase
CA: Constitutively activated
Endo-MT: Endothelial-to-mesenchymal transition
EMT: Epithelial-to-mesenchymal transition
ASP-RNAi: Allele-specific RNAi
mrSC: Muscle-residing stroma cell
NSAIDS: Nonsteroidal anti-inflammatory drugs
CTX: Cardiotoxin
TNFα: Tumor necrosis factor-α
